# Trust in Information Sources and Parents’ Knowledge, Attitudes, and Practices (KAP) of Children’s PCV13 Vaccination in the Yangtze River Delta Region, China

**DOI:** 10.3390/vaccines13090947

**Published:** 2025-09-04

**Authors:** Zhangyang Pan, Fan Liang, Shenglan Tang

**Affiliations:** 1 Global Health Research Center, Duke Kunshan University, No. 8 Duke Avenue, Kunshan 215316, China; 2 Division of Social Sciences, Duke Kunshan University, No. 8 Duke Avenue, Kunshan 215316, China; fan.liang@dukekunshan.edu.cn; 3 Duke Global Health Institute, Duke University, Durham, NC 27708, USA; shenglan.tang@duke.edu

**Keywords:** China, pneumococcal conjugate vaccines, trust, information sources, parents, knowledge, attitude, practice

## Abstract

**Background:** Trust in information sources is essential to enhance an individual’s understanding of the message and boost their willingness to change or act on specific health behavior, including vaccine uptake. This study explores the association between trust in information sources and parents’ knowledge, attitudes, and practices regarding their children’s 13-valent pneumococcal conjugate vaccine (PCV13) uptake across seven cities in the Yangtze River Delta (YRD) region in China. **Methods:** A cross-sectional web-based survey was conducted from May to June 2023. Adult parents (N = 1304) who had at least one child aged 24 months or less and lived in the YRD region were recruited. The Adjusted Ordinary Least Squares (OLSs) regression model was applied to estimate the association between participants’ level of trust in different information sources and their knowledge, attitudes, and practices of children’s PCV13 vaccination. **Results:** Information from the Disease Control and Prevention Center (CDC) source received the highest trust score. Age, gender, education, and annual household income were related to varied trust levels in specific sources. Trust in the health service provider source was significantly associated with a better command of PCV13 knowledge, acceptance of PCV13, and a higher likelihood of vaccination. Trust in online community sources was positively associated with vaccine uptake. **Conclusions:** The study participants highly trusted information from health service provider sources. These sources may be effective channels with potential to enhance parents’ vaccine knowledge and acceptance of PCV13. Public health workers could utilize trusted sources to disseminate the benefits of the PCV13 and encourage the uptake of the vaccine.

## 1. Introduction

Pneumococcal diseases caused by Streptococcus pneumoniae remain a major public health threat worldwide. China was among the 10 countries with the highest mortality of pneumococcal death in children under five years old in 2000 [1]. Recent data showed that annual pneumococcal deaths in China had dropped by 49% from 15,600 (uncertainty range: 10,800–17,300) to 8000 (5500–8900), while unevenly distributed disease burdens remained across different regions [2]. The World Health Organization (WHO) recommends that 10 vaccines or antigens be included in all immunization programs, one of which is the pneumococcal conjugate vaccine (PCV) [3]. Implementing PCV into routine childhood immunization schedules has been proven to significantly reduce invasive pneumococcal disease (IPD) and its related hospitalization [4].

In 2008, 7-valent PCV was launched in China and then replaced by 13-valent in 2016. However, the China National Immunization Program (NIP) has not yet included PCV13 as a routine vaccine for children. Therefore, PCV13 remains an optional non-NIP vaccine, requiring out-of-pocket payment by the children’s caregivers. Due to the non-mandate feature, high expenditure (Around USD68.12 per dose in 2021), and low awareness, the estimated 2021 three-dose PCV13 coverage was 16.13% among children under five years in nine provinces across China, much lower than the global average of 48% [5]. A scoping review indicates the major barriers to increasing the uptake of non-NIP vaccines in China include five main factors, namely, access, affordability, awareness, acceptance, and activation, of which interpersonal and media influence may affect vaccine acceptance when people obtain non-NIP vaccine information through various sources [6].

Trust enables the function of society as it reduces the complexities of collaboration and increases communication effectiveness. Trust could also be directed to vaccines. Among multiple determinants of vaccination, trust in individuals, institutions, and the whole system stands out as an underpinning factor. Larson argued that trust is the utmost factor in vaccine acceptance compared to any piece of information [7]. An individual accepts information partly based on trust in information sources. The more trust a source is perceived to have, the higher the likelihood that the information receiver would accept the information the source delivers [8].

Moreover, parents are usually the decision makers for children’s PCV13 vaccination [9]. While policy-shaping efforts are underway to introduce PCV13 into the NIP, increasing parents’ awareness and acceptance of PCV13 could be a short-term intervention to enhance vaccine coverage and reduce pneumococcal disease burdens. Knowledge, attitudes, and practices (KAPs) are valuable indicators in public health research to monitor and predict people’s health behaviors [10]. The previous literature suggests that trust in information sources is essential to enhance an individual’s understanding of the message and boost their willingness to change or act on specific health behaviors, including vaccine uptake [11,12]. During the COVID-19 outbreak, researchers in China, the United Arab Emirates (UAE), and the United States examined trust in information sources and their relations with the suggested protective behaviors. Other vaccine studies addressed the exposure to information sources and their association with parents’ vaccine beliefs, knowledge, and the children’s vaccination status regarding different types of pediatric vaccines [13,14,15,16]. New media and web-based platforms form a dynamic information environment in China, fundamentally shaping people’s information-seeking patterns. Along with the evolution of information sources, how trust in these sources influences parents’ knowledge, attitudes, and vaccination decision-making for non-NIP vaccines remains unknown.

This study aims to investigate the relationship between the demographic and socio-economic characteristics of study participants from the YRD region and their trust levels in various information sources. It also explores the association between trust in different information sources and parents’ knowledge, attitudes, and practices regarding children’s PCV13 vaccination. The findings would provide evidence and might shed light on more effective vaccine information dissemination strategies for public health workers.

## 2. Materials and Methods

### 2.1. Study Setting and Sample

A cross-sectional, web-based survey was conducted from 10th May to 7th June 2023, through Weidiaocha (Zhongyue Online Technology Co., Ltd., Qingdao, Shandong), a professional survey platform with a non-governmental background in China (www.weidiaocha.com, accessed on 10 May 2023). The platform has been used in other social science research [17,18]. Similar online platforms, such as Wenjuanxing, have also been widely applied in public health studies to collect population information efficiently [19,20]. Eligible participants were adult parents (18 years and older) with at least one child aged less than 24 months (including 24 months) and who lived in seven cities of the YRD region, namely, Shanghai, Wuxi, Suzhou, and Nanjing from Jiangsu Province, and Hangzhou, Ningbo, and Shaoxing from Zhejiang Province, by the time of the study.

The YRD region is China’s most developed economic cluster. The area encompasses Shanghai and part of Jiangsu, Zhejiang, and Anhui provinces, contributing approximately a quarter of China’s GDP [21]. The first-dose PCV13 vaccine coverage in Zhejiang, Shanghai, Anhui, and Jiangsu was 59.57%, 50.16%, 46.96%, and 43.95%, respectively, which were top of the nationwide coverage ranking [5,22]. Sufficient healthcare resources, good vaccine supply, cold chain infrastructure, and the high willingness of parents in this region to pay for the non-NIP vaccines mitigate many barriers to vaccine coverage [23,24,25]. Thus, we could explore the influence of trust in information sources on caregivers’ KAP of PCV13 vaccination. The seven cities were chosen based on the 2022 annual per capita disposable income. All cities exceeded the 65,000 Chinese Yuan Renminbi (CNY; approximately USD9155 by the 2023 average USD-CNY exchange rate of 7.1) threshold.

The survey platform, Weidiaocha, owns a database of more than 10 million samples across over 20 provinces in China by the time the study began. It reports 2.5 million active users in its dedicated online platform matrix, including apps, websites, and WeChat. Weidiaocha was chosen as the primary recruitment setting for several reasons: (1) their database was formed by a voluntary-based registration through multiple online and offline channels, (2) 93.2% of Weidiaocha’s samples are in the age group of 20 to 49 who are our primary target population group, and (3) previous research using this recruitment method to sample parents among the specific population. Cohen’s statistical power analysis using a moderate effect size and power of 0.95 was utilized to calculate the sample size for this study.

### 2.2. Data Collection

We recruited 100 participants via Weidiaocha for the pilot run of the survey before its formal launch. This is to check the Weidiaocha platform’s effectiveness and validity in participant recruitment, as well as testing the survey question responsiveness of our target population group. Since no survey question was revised after the pilot phase, we included the pilot-run data in the final study analysis. The survey invitation was distributed through email, WeChat, and app notice to individuals whose registered information met the study’s criteria by location and age. People who were interested in participating in the survey could click the link. After providing their consent, they could begin to answer the questionnaire. The individual participant’s IP address was recorded in the system and used to verify the location criteria. Parental status and the participant’s youngest child’s age were required to be filled out as two filtering questions before answering the rest of the survey questions.

**Figure 1:**
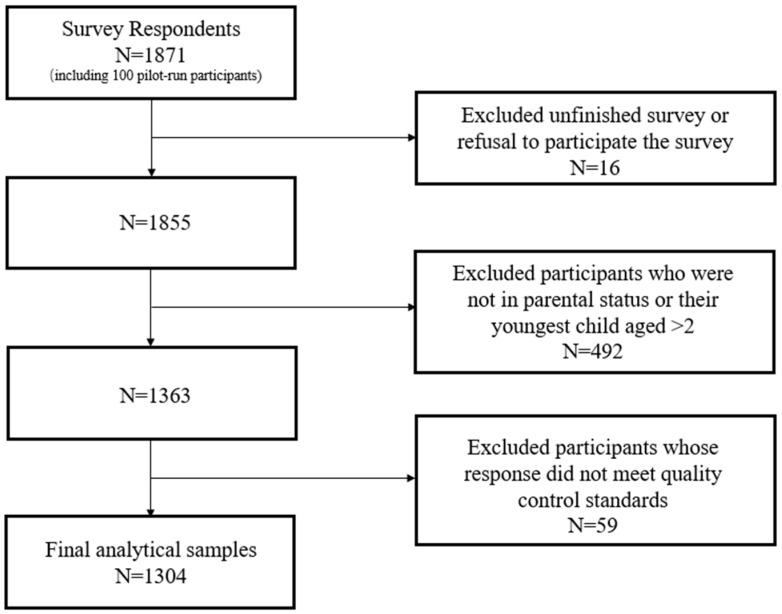
Analytic sample flow chart.

A total of 1871 respondents opened the survey link. Among the 1855 completed surveys, we excluded 492 participants who did not meet our study enrollment criteria. We also applied attention filter questions and ‘trap’ questions as standard quality control for the survey. One of the attention filter questions asked of the respondents was “What is the number of hours in a day?” and the options given were “[1] 14; [2] 20; [3] 24; [4] 30”. Traps were set for the options of different questions. For example, respondents who chose ‘Yes’ in children’s PCV13 vaccination status but answered ‘never heard of PCV before’ in the information source question will be defined as not passing the quality control check. Other quality control measurements included the similarity of trust level grading toward all information sources and predominant ‘I am not sure’ options in the knowledge section. Therefore, we excluded 59 completed surveys after applying these measures. Our final analytic sample included 1304 respondents (Figure 1).

### 2.3. Measurement

The questionnaire was designed in accordance with the China Family Panel Studies and the Expert Consensus on Immunoprophylaxis of Pneumococcal Disease (2020 version), as well as previous research on the association between information source use and vaccination. The final version was also adjusted based on insights from experts in health communication and public health policy [12,26,27,28,29]. The study questionnaire included three major parts: (1) demographic and socio-economic information; (2) sources of vaccine information and levels of trust in different sources; and (3) knowledge, attitudes, and practices (KAP) of PCV13 vaccination.

Participants’ demographic and socio-economic information was collected, and they were referred to several recent pieces of published literature about parents’ vaccine acceptance and willingness to pay for non-NIP vaccines in China [9,15,24,25]. The information includes age (18–24, 25–34, 35–45, >45), gender (female and male), annual household income in different ranges (<20,000 CNY, 20,000~49,999 CNY, 50,000~99,999 CNY, 100,000~300,000 CNY, >300,000 CNY), education attainment (secondary school and below, high school and technical secondary school, junior college, Bachelor’s degree, postgraduate and above), and the number of children (1, or >1). Children’s PCV13 vaccination status was also collected.

Sources of information combined the categories used in previous COVID-19 vaccination studies [15,30,31]. A multiple-choice question was provided about which source the participants had used to obtain information about PCV13. The participants were required to choose based on their information-seeking and exposure experiences. Nine sources were listed: CDC, vaccination clinics, hospitals, medical media, official media, family/friends, maternal apps, online forums, and social media. The last option is addressed as ‘I never heard about PCV13′ to filter participants unaware of the vaccine. Then, respondents were asked to rate the trust level for each of the nine information sources. As a mental status, trust could be graded by the subjective certainty of the pertinent beliefs rather than generating a simple binary outcome [32]. Therefore, a Five-Point Likert scale of trust level was set for the rating, ranging from [1] distrust at all, [2] somewhat distrust, [3] neutral, [4] somewhat trust, to [5] full trust.

The primary outcomes were parents’ knowledge, attitudes, and practices regarding their children’s PCV13 vaccination. The PCV13 knowledge was designed according to the 2020 version of the expert consensus on immunoprophylaxis of pneumococcal disease [27]. There were six narratives covering the vaccine category, vaccine schedule, the benefits of PCV13 vaccination, and the knowledge of potential adverse effects after the injection. Parents were required to evaluate the correctness of the narrative by a Five-Point Likert scale: [1] wrong, [2] maybe wrong, [3] I am not sure, [4] maybe correct, and [5] correct. The answers of ‘maybe correct,’ ‘I am not sure,’ and ‘maybe wrong’ were combined into a single category of ‘uncertain’. All narratives are correct based on the administered guidelines and the expert consensus. These six questions are averaged to construct an index of parents’ knowledge (Cronbach’s α = 0.68, M = 2.44, and SD = 0.35).

Parental attitudes toward PCV13 were assessed through a general attitude narrative—‘In all, I am hesitant to vaccinate my child with PCV13.’ A Five-point Likert scale was applied to evaluate the attitudes. Respondents were required to choose [1] disagree, [2] somewhat disagree, [3] neutral, [4] somewhat agree, and [5] agree for each narrative (M = 2.59, SD = 1.21).

PCV13 vaccination practice was asked through two separate questions. Parents were first asked whether or not their child had been PCV13 vaccinated. Those who responded ‘Yes’ will end the survey. For those who answered ‘No’ to the first question, a follow-up question was asked about their children’s PCV13 vaccination plan. Respondents were required to choose one of the four options: [1] No vaccination plan; [2] Not for now, but I will seek further information; [3] Plan to, but I haven’t made an appointment; and [4] I’ve already made a vaccination appointment. Those vaccinated were categorized into [5] Vaccinated (M = 4.07, SD = 1.31). Details of the questionnaire are provided in Supplementary Material (Table S1).

### 2.4. Statistical Analysis

Quantitative analyses were performed using STATA/SE version 17 (Stata Corp, College Station, TX, USA). Primarily, descriptive statistics analysis was conducted to summarize the participants’ demographic and socio-economic characteristics, sources of information used, levels of trust in different sources, parents’ knowledge, vaccine hesitancy, and vaccination plan. Frequencies, proportions, and means with standard deviations were applied to the descriptive data display.

By extending the definition to the paradigm of trust in analytic philosophy, Freiman divided trust into interpersonal and institution-based trust [33]. Interpersonal trust is “an attitude we have toward people whom we hope will be trustworthy” [34]. As we also gain information from collective sources, trust can be directed to the attitudes toward institutional entities, such as government, healthcare institutions, commercial entities, and social groups [35]. Based on the theories and to increase the statistical power of the analysis, information sources were combined into source clusters. CDC, vaccination clinics, hospitals, and medical media were grouped as the ‘Medical institutions’ group (Cronbach’s α = 0.77, M = 4.93, and SD = 0.62). Maternal apps, online forums, and social media were grouped into ‘Online community’ (Cronbach’s α = 0.81, M = 2.94, and SD = 0.72). Official media stands as an independent source type (M = 4.18, SD = 0.81). Family and friends represent the participants’ personal network source (M = 3.63, SD = 0.78).

Ordinary Least Squares (OLS) regression was applied to analyze the influencing factors of trust in different information sources. As evidence shows, age, gender, household income, and education level could potentially influence parents’ awareness and vaccine uptake practices. A recent study also showed that the number of children might dilute the family’s economic resources to spend on non-NIP vaccine expenditures [36]. Therefore, we included the age, gender, annual household income, education level, and number of children as independent variables. Furthermore, adjusted OLS regression models were used to estimate the effect of trust in information source groups on the three primary outcomes—knowledge, attitudes, and practices, with age, gender, household income, education level, and number of children as covariates. The statistical differences were considered significant when the *p*-value was <0.05.

## 3. Results

The demographic and socio-economic characteristics of the respondents of the web-based survey are reported in Table 1. Of the 1304 participants, 749 were female (57.4%). Overall, 77.6% are aged 25 to 34. More than half of the participants had higher education degrees, with 61.35% obtaining a bachelor’s degree or above. Overall, 70.2% reported annual household income ranging from CNY100,000 to 300,000 (USD14,084 to USD42,254, by the 2023 average USD-CNY exchange rate of 7.1). In total, 922 participants (70.7%) reported only having one child.

Table 2 illustrates the results of information sources the parents had used or been exposed to and the level of trust in different information sources. Vaccine clinic was reported by the largest proportion of participants (N = 712, 54.9%), followed by hospitals (N = 625, 48.2%) and family or friends (N = 614, 47.3%). By ranking the trust levels in different information sources, the CDC received the highest trust score of 4.48 out of 5 (SD = 0.71). Other sources that received over the 4.0 trust mark were official media (mean = 4.18, SD = 0.81), vaccination clinics (mean = 4.16, SD = 0.81), and hospitals (mean = 4.04, SD = 0.82). The lowest trust scores were found in social media sources (mean = 2.73, SD = 0.88).

**Figure 2:**
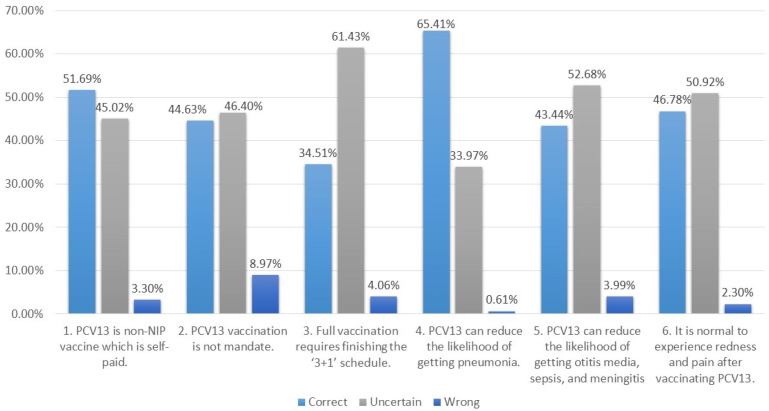
Participants’ PCV13 knowledge.

Parents’ PCV13 knowledge, as displayed in Figure 2, appears to have varied results across the six different narratives. The highest proportion of correct responses (65.4%) was found for Narrative Four of ‘PCV13 can reduce the likelihood of getting pneumonia’. The highest uncertainty (61.4%) was shown in Narrative Three about the PCV13 vaccination schedule. Only 34.5% of parents put a ‘Right’ response in this narrative. Compared with the result of Narrative Four, Narrative Five on PCV13’s efficacy to prevent otitis media, sepsis, and meningitis had a higher uncertainty (52.7%). All narratives showed less than a 10% rate of ‘Wrong’ response, indicating our study participants’ low prevalence of being misinformed on PCV13. The highest ‘Wrong’ (9.0%) was found in Narrative Two, showing that some parents might consider PCV13 as a mandatory vaccine.

Only 6.6% of the parents agreed with the statement that ‘Overall, I am hesitant to vaccinate my child with PCV13′. Overall, 46.3% of parents disagreed or partially disagreed with this statement. About one-third (30.7%) of parents stayed neutral. In total, 841 participants (64.5%) confirmed their children’s PCV13 vaccination status, with 20.6% reporting ‘plan to’ and 12.8% ‘Not for now, but I will seek further information’.

Table 3 shows the association between participants’ demographic and socio-economic factors and the level of trust in different health information sources among our study participants. In general, as age increased, participants showed a 0.07 decrease in trust in information from their personal network (*p* < 0.05). Compared with females, males have significantly lower trust in personal networks and online communities (*p* < 0.001). With the increase in annual household income, a 0.10 increase in trust in the online community was identified. Education was associated with a 0.06 increase in official media trust (*p* < 0.05) and a 0.09 increase in personal network trust (*p* < 0.001).

The association between trust in information sources and knowledge is demonstrated in Table 4. The adjusted results showed that parents’ knowledge level was associated with trust in the medical institution’s source. For every 1-point increase in trust score, parents’ knowledge score will increase by 0.08 (*p* < 0.001). No significant association was shown between trust in other source clusters and the knowledge of PCV13. Moreover, trust in medical institutions was related to lower parental vaccine hesitancy (*p* < 0.001). Trust in other information source clusters did not show a significant difference in the outcome of attitudes.

As for the vaccination practices, parents who placed higher trust in medical institutions (*p* < 0.01) and online community sources (*p* < 0.001) were found to be more positive in PCV13 vaccine uptake. However, trust in official media had a negative impact on vaccine uptake (*p* < 0.01). The table also lists the association between the control variables and the primary outcomes. With the increase in parents’ age, there was a higher likelihood of not vaccinating their children with PCV13. Male participants had lower PCV13 knowledge scores than females but were more likely to vaccinate their children with PCV13. Increased education levels would reduce parents’ PCV13 vaccine hesitancy and increase their likelihood of following the vaccination plan.

## 4. Discussion

The study examines the relationship between demographic and socio-economic factors and parents’ trust in different information sources. Secondly, it looked into parents’ trust in information sources and their knowledge, attitudes, and practices regarding children’s PCV13 vaccination. To do this, the study includes three focuses: (1) levels of trust in different vaccine information sources, (2) the demographic and socio-economic factors associated with the level of trust in different information sources, and (3) the association between the level of trust in different information sources and parents’ KAP regarding children’s PCV13 vaccination.

This study has several notable highlights. It is the first study in China to explore the association between trust in information sources and parents’ KAP regarding children’s PCV13 vaccination. It provides new insights for public health workers, who may consider strategically using this evidence to effectively disseminate vaccine information through trusted sources, thereby increasing vaccine uptake. Secondly, it provided more detailed descriptions about the sources in the survey rather than using a general media type (e.g., TV, newspaper, websites), which was more relevant to parents’ vaccine information-seeking practices.

The results of the quantitative analysis suggest a general higher trust in medical institution sources and official media sources and a lower trust in participants’ personal networks and online communities. The higher trust in the ‘medical institution’ sources among parents for pediatric vaccine information was in line with previous studies in Taiwan, China, and Mainland China [37,38]. Similar results were also shown in a western German study about COVID-19 vaccination, in which the adult population placed higher trust in health authorities and medical professionals, with social networks having the lowest trust [39]. The proportion of parents using each source did not show exact consistency with the trust level ranking. Despite expressing comparatively lower trust in the online community and information from personal network sources, the frequency of exposure to the two types of sources remains high. The paradox was consistent with the COVID-19 study in the UAE and Germany, where the sources with higher usage frequencies were not viewed as more trustworthy or vice versa [28,39]. A potential explanation would be the convenience of accessing information through social media. Using social media platforms to browse vaccine information was considered time-saving and efficient, as posts from individual and verified medical sources could be checked at the same time. It might also relate to the over-representativeness of female participants, as mothers tended to obtain more information from social media platforms and personal networks than their male counterparts [28].

Regarding the knowledge command level of PCV13, the survey results revealed high uncertainty among parents’ knowledge of PCV13. Over half of the participants were unsure about the vaccination schedule, PCV13′s efficacy in protecting children from meningitis, otitis media, other infections, and the adverse effects of vaccination. It may be partially due to the time that has passed since the scheduled vaccination. In addition, the widespread use of digital tools for vaccination appointment booking and reminders in the YRD region also saved parents the effort of remembering the details about PCV13, including the vaccine schedule and the total number of doses. The results also presented low rates of ‘wrong’ responses in each knowledge narrative, which indicated a high probability that very few of our study participants were misinformed about PCV13. Overall, 23% of parents reported being hesitant or somewhat hesitant about having their children vaccinated with PCV13. The proportion was closer to the findings in a study in Shanghai in which about 24.5% of parents had no intention of vaccinating their children with PCV13 [9]. When comparing the vaccination rate results with the population-based study in the Songjiang District of Shanghai, our study participants reported a higher vaccination rate (64.5%) than the PCV13 first dose coverage in Shanghai (54.7%, 2020) [40]. Considering the proportion of higher education and the annual household income level of our study participants, the higher vaccination rate was reasonably expected. The survey results also revealed that male participants scored lower on knowledge but reported higher rates of vaccination uptake. A potential explanation is that in China, male caregivers frequently act on the mother’s instructions to take children for vaccinations. Consequently, their role may be perceived more as the executor of the task rather than an engaged participant in knowledge acquisition. Further research is needed to explore the gender-based division of roles in vaccination decision-making and implementation.

Moreover, age, gender, and education were significantly associated with trust in specific sources, as indicated by the survey data. In this study, age was found to be associated with lower trust in personal network sources. Previous studies only confirmed age’s association with different information sources using habit [28]. Women placed higher trust in social media, and people with higher education attainment trusted official media and close social networks more, which is similar to previous studies [28,41]. People with higher education attainment were found to have higher trust in family and friends’ sources, which was also identified from the UAE study [28]. A possible explanation is that these individuals may have a higher likelihood of close contact with people who have medical backgrounds. This group of people was also found to have higher trust in online community sources, which might be explained by the correlation between education and health literacy in previous evidence—specifically, people with higher education backgrounds tend to be more competent in navigating credible and reliable information from online communities [42].

More importantly, the study results showed that trust in medical institution sources was related to better PCV13 knowledge, less vaccine hesitancy, and more positive vaccine uptake practices. Trust in other information source clusters did not show similar consistency in the association with the three primary outcomes. The findings were in line with two U.S.-based studies. One reported trust in medical institution sources was related to lower vaccine hesitancy [43], while the other shows trust in the CDC as a source of information on vaccines was a strong predictor of influenza vaccination [44]. Previous studies also showed that participants who reported being exposed to information from the Ministry of Health, physicians, and pediatricians had higher vaccine knowledge scores [45,46]. To our surprise, trust in official media sources was negatively associated with vaccine uptake. One potential explanation would be the low frequency of use of official media among parents as a source of vaccine information, as many of them seldom provide specific information about PCV13 targeting caregivers. Therefore, parents who reported high trust in official media were unable to obtain sufficient information about PCV13. Their less informed status might reduce the likelihood of their children’s vaccine uptake.

Moreover, a significant association was found between the trust of online community sources and vaccine uptake practices. However, previous studies in China have yielded inconsistent findings regarding the relationship between trust in online media sources and vaccine uptake. Two studies confirmed the promoting effect of social media on COVID-19 or influenza vaccination [15,29]. Another study found no direct influence of media credibility on influenza vaccination [47]. As for the case of PCV13 vaccination, one possible explanation could be that the parents’ decision-making was highly reliant on other people’s previous practices. This might also suggest why trust in online community sources had no association with PCV13 knowledge and attitudes, yet did with the action of vaccine uptake. The results could also be partially explained by the social media health content regulation in China, which had mitigated the risk of misinformation and enhanced health behaviors. The major social media platforms all apply such regulations. For instance, doctors running accounts on WeChat, Xiaohongshu, and Weibo were required to provide a professional certificate for account verification before posting medical suggestions.

The present study has several limitations. The cross-sectional nature of the web-based survey does not allow for the establishment of causal inference. Recruitment of the participants from the Weidiaocha platform’s database was opportunistic and self-voluntary, which may introduce sampling bias and limit the generalization of our findings. The survey information was self-reported and might be subject to information bias. Finally, the study was conducted right after the World Health Organization declared the end of the COVID-19 pandemic, which may have influenced the general trust evaluation and parents’ awareness of vaccine topics periodically.

## 5. Conclusions

Based on our study findings, the CDC was the most trusted information source by our study participants. Parents’ trust in other sources might be subject to age, gender, education, and annual household income levels. Trust in the medical institution sources was associated with a better command of PCV13 knowledge, reduced vaccine hesitancy, and increased the likelihood of vaccine uptake, even after adjusting for the effects of demographic and socio-economic factors. The significant influence of online communities on driving the vaccination uptake may be utilized for vaccine awareness-raising campaigns. Public health workers could utilize trusted sources to disseminate information about PCV13 and encourage the uptake of the vaccine.

## Figures and Tables

**Table 1:** The demographic and socio-economic characteristics of the web-based survey participants (N = 1304).

Variable		N (%)
Age	18–24	79 (6.1%)
	25–34	1012 (77.6%)
	35–45	171 (13.1%)
	>45	42 (3.2%)
Gender	Female	749 (57.4%)
	Male	555 (42.6%)
Education attainment	Secondary school and below	56 (4.3%)
	High school and technical secondary school	159 (12.2%)
	Junior college	289 (22.2%)
	Bachelor’s degree	719 (55.1%)
	Postgraduate and above	81 (6.2%)
Annual household income (Unit: CNY)	<20,000	0 (-)
	20,000 to 49,999	20 (1.5%)
	50,000 to 99,999	230 (17.6%)
	100,000 to 300,000	915 (70.2%)
	>300,000	139 (10.7%)
Number of children	1	922 (70.7%)
	>1	382 (29.3%)

**Table 2:** Participants’ vaccine information sources and their levels of trust in different information sources (N = 1304).

Category	Sources	Trust Level (Mean, SD)	Frequency
Medical institutions	CDC	4.48 (0.71)	497
	Vaccination clinics	4.16 (0.81)	712
	Hospitals	4.04 (0.82)	625
	Medical media	3.68 (0.86)	328
Official media	Official media	4.18 (0.81)	476
Personal network	Family/friends	3.63 (0.78)	614
Online community	Maternal Apps	3.28 (0.91)	358
	Online forum	2.85 (0.89)	431
	Social media	2.73 (0.88)	198

**Table 3:** OLS regression results of influencing factors of trust in different information sources.

	Trust in Different Information Source Categories							
Variables	Medical Institutions		Official Media		Personal Network		Online Community	
	SMD (β)	SE	SMD (β)	SE	SMD (β)	SE	SMD (β)	SE
Constant	4.03 ***	(0.13)	3.90 ***	(0.18)	2.82 ***	(0.16)	2.98 ***	(0.17)
Age	−0.05	(0.03)	−0.05	(0.04)	−0.07 *	(0.04)	0.04	(0.04)
Male	−0.06	(0.03)	0.04	(0.05)	−0.17 ***	(0.04)	−0.16 ***	(0.04)
Annual household income	0.03	(0.03)	0.04	(0.04)	0.01	(0.04)	0.10 *	(0.04)
Education	0.03	(0.02)	0.06 *	(0.03)	0.09 ***	(0.02)	0.08 **	(0.02)
Number of children	−0.02	(0.04)	0.05	(0.05)	0.01	(0.05)	−0.02	(0.05)
R2	0.01		0.01		0.03		0.03	

* *p* < 0.05, ** *p* < 0.01, *** *p* < 0.001.

**Table 4:** Adjusted OLS regression results of influencing factors of parents’ knowledge, attitudes, and practices regarding children’s PCV13 vaccination.

		Knowledge		Vaccine Hesitancy		Vaccine Uptake	
		SMD (β)	SE	SMD (β)	SE	SMD (β)	SE
Constant		1.94 ***	(0.01)	4.67 ***	(0.35)	2.58 ***	(0.38)
Predictors	Medical institutions	0.08 ***	(0.02)	−0.49 ***	(0.07)	0.24 **	(0.07)
	Official media	0.01	(0.01)	0.07	(0.05)	−0.11 **	(0.05)
	Personal network	−0.01	(0.01)	−0.03	(0.05)	0.02	(0.05)
	Online community	0.02	(0.01)	−0.05	(0.05)	0.21 ***	(0.06)
Covariates	Age	0.01	(0.02)	0.11	(0.06)	−0.22 ***	(0.06)
	Male	−0.08 ***	(0.02)	0.03	(0.07)	0.23 **	(0.07)
	Annual household income	0.02	(0.04)	−0.03	(0.06)	0.01	(0.07)
	Education	0.01	(0.01)	−0.08 *	(0.04)	0.08 *	(0.04)
	Number of children	0.02	(0.02)	0.09	(0.07)	0.03	(0.08)
R2		0.05		0.08		0.05	

* *p* < 0.05, ** *p* < 0.01, *** *p* < 0.001.

## Data Availability

The participants of this study have consented to their data being used for research purposes only. The datasets generated and/or analyzed during the study are not publicly available but can be accessed by contacting the corresponding author on reasonable request.

## Supplementary Materials

The following supporting information can be downloaded at https://www.mdpi.com/article/10.3390/vaccines13090947/s1: Table S1: Information sources and parents’ knowledge, attitudes, and practices (KAP) of children’s PCV13 vaccination—Survey Questionnaire.

## Abbreviations

The following abbreviations are used in this manuscript:

CDC	Center of Disease Control and Prevention
IPD	Invasive pneumococcal disease(s)
KAP	Knowledge, attitudes, and practices
NIP	National Immunization Program
OLS	Ordinary Least Square
PCV	Pneumococcal conjugate vaccine
UAE	United Arab Emirates
YRD	Yangtze River Delta
