# Supplementary material for: Trust in Information Sources and Parents’ Knowledge, Attitudes, and Practices (KAP) of Children’s PCV13 Vaccination in the Yangtze River Delta Region, China
Source: Vaccines (Basel). 2025 Sep 4;13(9):947. doi: 10.3390/vaccines13090947 (PMC12474068; doi:10.3390/vaccines13090947)
Supplement: Supplementary file 1 [file vaccines-13-00947-s001.zip › vaccines-3788617-Table S1.pdf]

Information sources and parents’ knowledge, attitudes, and practices (KAP) of children’s PCV13 vaccination

## Survey Questionnaire

| Demographic and socio-economic Information |
|---|
| 1. Gender: [1] Female [2] Male |
| 2. Age: [1] 18-24; [2] 25-34; [3] 35-45; [4] >45 |
| 3. Your education attainment:<br>[1] secondary school and below;<br>[2] High school and technical secondary school;<br>[3] Junior college;<br>[4] Bachelor degree;<br>[5] Postgraduate and above. |
| 4. Estimated household income in the past 12 months (Unit: CNY):<br>[1] <20,000; [2] 20,000 to 49,999; [3] 50,000 to 99,999; [4] 100,000 to 300,000; [5] >300,000 |
| 5. Single child family:<br>[1] Yes; [2] No |
| **Information sources** |
| 6. Which sources do you get PCV13 information? (multiple selection)<br>[1] CDC;<br>[2] Vaccine clinics;<br>[3] Hospitals;<br>[4] Medical media;<br>[5] Official media;<br>[6] Family/Friends;<br>[7] Maternal apps;<br>[8] Online forum (Tieba, WeChat Group);<br>[9] Social media (Xiaohongshu, Weibo etc.);<br>[10] Never heard of PCV13 before. |
| 7. Rate your trust levels in the sources listed (same as Q6) by Five-Point Likert scale:<br>[1] distrust at all; [2] somewhat distrust; [3] neutral; [4] somewhat trust; [5] fully trust |
| **PCV13 Knowledge** |
| Rate true/wrong for the six narratives about PCV13 using a Five-Point Likert scale:<br>[1] wrong; [2] maybe wrong; [3] I am not sure; [4] maybe correct; [5] correct. |
| 8-1. PCV13 is a non-NIP vaccine which is self-paid. |
| 8-2. PCV13 vaccination is not mandate. |
| 8-3. Full vaccination requires finishing the ‘3+1’ schedule. |
| 8-4. PCV13 can reduce the likelihood of getting pneumonia. |
| 8-5. PCV13 can reduce the likelihood of getting otitis media, sepsis, and meningitis. |
| 8-6. It is normal to experience redness and pain after vaccinating PCV13. |
| **Attitudes towards PCV13** |
| Rate agree/disagree for the narratives about PCV13 vaccination using a Five-Point Likert scale: |
| 9. In all, I am hesitant to vaccinate my child with PCV13. |

| |
|---|
| [1] disagree, [2] somewhat disagree, [3] neutral, [4] somewhat agree, [5] agree |
| **PCV13 vaccination plan** |
| 10. Has your youngest child been vaccinated with PCV13? [1] Yes; [2] No.<br>(Answer 'Yes' will end the survey) |
| 11. What is your plan for your children's PCV13 vaccination?<br>[1] No vaccination plan;<br>[2] Not for now, but I will seek further information;<br>[3] Plan to, but I have not made an appointment;<br>[4] I have already made a vaccination appointment. |
